# Esophageal atresia with tracheo-esophageal fistula: Accidental transtracheal gastric intubation

**DOI:** 10.4103/0971-9261.59608

**Published:** 2009

**Authors:** N. N. Hombalkar, Sara Dhanawade, Priya Hombalkar, Dhananjay Vaze

**Affiliations:** Department of Surgery, Govt. Medical College, Miraj, Maharashtra, India; 1 Department of Pediatrics, Bharati Vidyapeeth Medical college, Wanlesswadi, Miraj, Maharashtra, India

**Keywords:** Esophageal atresia, tracheo-esophageal fistula

## Abstract

The diagnostic feature of esophageal atresia (EA) is the inability to pass a catheter into the stomach. EA can be ruled out if the feeding tube can be passed into the stomach. In EA, when a tracheo-esophageal fistula (TEF) is present, theoretically the feeding tube can find its way into the stomach via tracheal route and through the TEF. We report such a rare occurrence. In this situation, the diagnosis and further management of EA and TEF was delayed.

## INTRODUCTION

The diagnostic feature of esophageal atresia (EA) is the inability to pass a catheter into the stomach. EA can be ruled out if the feeding tube can be passed into the stomach. Many a times, the neonates with EA and TEF are first diagnosed by neonatologists, and then they are referred for surgical correction to pediatric surgeons.

## CASE REPORT

**Figure 1:**
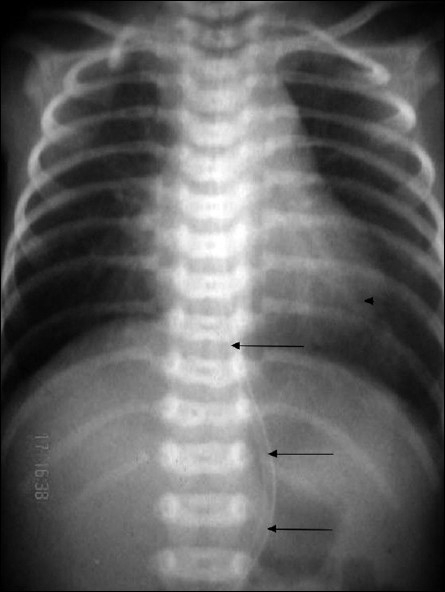
Photograph showing passage of nasogastric tube into stomach in a case of esophageal atresia with tracheo-esophageal fistula.

A 16-hour-old female child (birth weight 1400 g), born to parents with second-degree consanguineous marriage, presented with an antenatal suspicion of esophageal atresia (EA). Respiratory distress, excessive salivation, and abdominal distension were noticed. No other congenital anomalies were detected at birth. A feeding tube was passed to confirm the antenatal suspicion of EA and tracheo-esophageal fistula (TEF). This tube could be passed into the stomach. The position of the tube was confirmed on abdominal radiograph [Figure 1]. As this ruled out EA, the patient was further managed for respiratory distress with the feeding tube in place. After a few hours, on strong clinical suspicion of EA with TEF, the feeding tube was removed. Subsequent attempts at passing a feeding tube into the stomach were unsuccessful. The radiographs were repeated now with a 10F stiff red rubber catheter. The catheter could be seen arrested in the blind upper esophageal pouch, which was located fairly high up in the neck. The patient was then transferred to our institute for surgical intervention. The patient identity and all the radiographs obtained during the clinical course were cross checked and any kind of mistake was ruled out. A right lateral thoracotomy revealed an EA of type III b with a wide TEF. Upper blind pouch was very high up in the neck and could not be retrieved into the right chest for primary anastomosis. Thus a decision of fistula ligation, upper pouch cervical esophagostomy, and a gastrostomy was made. The upper pouch fistula was ruled out during cervical esophagostomy. A gastrostomy was also placed. The baby tolerated these procedures well but succumbed on post-operative day 3.

## DISCUSSION

Many a times, the neonates with EA and TEF are first diagnosed by neonatologists, and then they are referred for surgical correction to pediatric surgeons. In spite of classical teaching of passing a stiff red rubber catheter of 10F caliber through mouth, it is a routine practice to pass a 5F or 6F infant feeding tube through the nose for the diagnosis of EA. EA can be diagnosed if there is inability to pass the feeding tube into the stomach and radiographs show a coiled catheter in the upper esophageal pouch.[1] But rarely gastric position of the feeding tube in a case of EA with TEF can be obtained delaying the diagnosis of EA with TEF. Only one case has been reported so far in the literature.[2] In this situation, the feeding tube could reach the stomach from the upper pouch then into the tracheal route and then through the TEF. A peculiar pathological anatomy makes this occurrence possible. When the upper blind pouch is unduly short, the likelihood of the small caliber feeding tube easily recoiling and finding its way into the neighboring trachea is indeed high. With a wide TEF, the feeding tube that has entered the trachea can then travel down to the stomach easily. In these situations, diagnosis and management of EA and TEF can be delayed compromising the surgical outcome. This clinical situation can be avoided by using a stiff rubber catheter instead of a soft feeding tube for the diagnosis of EA and TEF.
